# Diagnostic Value of 18F-FDG PET/CT in Guiding Biopsy Decisions and Differentiating Infectious, Inflammatory and Malignant Lesions

**DOI:** 10.3390/jcm15062132

**Published:** 2026-03-11

**Authors:** Özlem Güler, Sonay Arslan, Zeynep Bayraktar, Oğuzhan Sözen, Birsen Mutlu, Sibel Balcı, Serkan İşgören, Sıla Akhan

**Affiliations:** 1 Department of Infectious Diseases and Clinical Microbiology, Faculty of Medicine, Kocaeli University, Kocaeli 41380, Türkiye; sonay.arslan.sonay@gmail.com (S.A.); drzeynepbayraktar@gmail.com (Z.B.); birmutlu@gmail.com (B.M.); cetinakhan@yahoo.com.tr (S.A.); 2 Department of Nuclear Medicine, Faculty of Medicine, Kocaeli University, Kocaeli 41380, Türkiye; 1997oguzhansozen1997@gmail.com (O.S.); serkanisgoren@hotmail.com (S.İ.); 3 Department of Biostatistics and Medical Informatics, Faculty of Medicine, Kocaeli University, Kocaeli 41380, Türkiye; b.s.balci@gmail.com

**Keywords:** 18F-FDG, PET CT scan, infection, inflammation, malignancy, biopsy, Mycobacterium infections

## Abstract

**Background/Objectives:** 18F-fluorodeoxyglucose (FDG) positron emission tomography/computed tomography (PET/CT) is used in oncology but has limited specificity due to uptake in infectious and inflammatory conditions. This study evaluated the diagnostic value of 18F-FDG PET/CT in patients with infectious, inflammatory, and malignant lesions and its role in guiding histopathological biopsy decisions in an infectious disease setting. **Methods:** A retrospective cohort study included 186 adult patients who underwent 18F-FDG PET/CT between 2018 and 2023 for diagnostic evaluation at a tertiary care hospital. Clinical indications were fever of unknown origin (FUO), inflammation of unknown origin (IUO), lymphadenopathy of unknown origin, and suspected solid mass. Diagnostic yield, biopsy decisions, and factors associated with biopsy were analyzed. **Results:** The diagnostic yield of 18F-FDG PET/CT was 58.6%, with higher in malignant conditions (hematologic malignancy 85.7%, solid organ malignancy 88.9%) and lower in autoimmune/inflammatory diseases (20.8%) and mycobacterial infections. PET/CT showed moderate sensitivity (59.8%) and high specificity (98.7%) for infection detection, improving to 67.8% sensitivity after excluding mycobacterial infections. Biopsy was performed more in patients with lymphadenopathy, higher SUVmax (>7.4), and PET/CT findings not suggestive of infection. Analysis identified lymphadenopathy (aOR = 2.77), PET/CT not suggestive of infection (aOR = 4.73), and SUVmax > 7.4 (aOR = 4.98) as predictors of biopsy. **Conclusions:** 18F-FDG PET/CT provides moderate diagnostic value across infectious, inflammatory, and malignant diseases and guides biopsies effectively, particularly in patients with lymphadenopathy, elevated SUVmax, and non-infectious findings. Its limited performance in mycobacterial and autoimmune diseases requires cautious interpretation. Overall, 18F-FDG PET/CT supports clinical decisions in complex diagnostic scenarios.

## 1. Introduction

18F-fluorodeoxyglucose (FDG) positron emission tomography/computed tomography (PET/CT) is a widely used diagnostic tool in oncology for both staging and evaluation of treatment response. It is also used in radiotherapy planning in advanced-stage malignancies [1]. Nevertheless, FDG uptake is not specific to malignant processes and may also be observed in benign infectious and inflammatory conditions. Activated neutrophils, macrophages, and lymphocytes demonstrate increased glucose metabolism similar to that of malignant cells; therefore, benign processes may mimic malignancy on 18F-FDG PET/CT [2].

Fever of unknown origin (FUO) and inflammation of unknown origin (IUO), which is a more recently described condition characterized by elevated inflammatory markers such as C-reactive protein (CRP) and erythrocyte sedimentation rate (ESR), present particularly challenging diagnostic scenarios. These conditions encompass a broad spectrum of underlying etiologies, including infections, non-infectious inflammatory diseases, malignancies, and other disorders. According to the European Association of Nuclear Medicine (EANM) Infection and Inflammation Committee consensus recommendations, 18F-FDG PET/CT should be performed after initial laboratory and radiological baseline investigations and exclusion of cardiac sources in patients meeting the established criteria [3]. Similarly, FDG PET/CT is frequently used to differentiate neoplastic conditions, such as lymphoma, from infectious and inflammatory causes in patients with unexplained lymphadenopathy [4]. In clinical practice, patients presenting with unexplained lymphadenopathy, fever, or persistent inflammation are frequently referred to Infectious Diseases clinics for further evaluation, in both inpatient and outpatient settings. Patients admitted to infectious disease clinics may present with highly heterogeneous clinical manifestations, and in many cases, establishing a definitive diagnosis may be prolonged or remain unresolved.

In such cases, 18F-FDG PET/CT is frequently used as a supportive diagnostic tool. This is because it allows for a whole-body evaluation in a single session. As a result, it may help shorten the diagnostic process and length of hospital stay [5]. Beyond its diagnostic contribution, 18F-FDG PET/CT plays a critical role in guiding histopathological biopsy decisions by identifying metabolically active target lesions. Although 18F-FDG PET/CT is a costly diagnostic modality, delays in establishing a definitive diagnosis may result in disease progression and increased mortality [6].

This study aimed to assess the effectiveness of 18F-FDG PET/CT in the diagnostic evaluation of these patients. Furthermore, the role of 18F-FDG PET/CT in guiding histopathological biopsy decisions was also evaluated. The study investigated the potential of 18F-FDG PET/CT to reduce unnecessary invasive procedures by identifying patients who truly require biopsy and the factors associated with the need for biopsy.

## 2. Materials and Methods

### 2.1. Study Design and Patient Population

This retrospective cohort study was conducted at a tertiary care university hospital between 2018 and 2023. The report was prepared in accordance with the Strengthening the Reporting of Observational Studies in Epidemiology (STROBE) statement, as recommended by the EQUATOR Network [7]. Adult patients (≥18 years) who underwent 18F-FDG PET/CT scans as part of a diagnostic evaluation at the Department of Infectious Diseases were included in the study.

Patients with a known diagnosis of malignancy, those with previously scheduled 18F-FDG PET/CT examinations for oncologic surveillance, and those who were lost to follow-up during the minimum six-month diagnostic evaluation period were excluded from the study. Patients who had undergone major surgery or experienced significant trauma within the preceding three months were also excluded due to the potential impact on FDG uptake [8]. Additionally, patients without at least six months of follow-up to establish a definitive diagnosis were excluded.

18F-FDG PET/CT is a costly diagnostic procedure that is requested for a relatively limited patient population in routine clinical practice. The clinical indications for 18F-FDG PET/CT are heterogeneous in patients evaluated by infectious disease specialists. Therefore, all eligible patients who met the inclusion criteria during the study period were included to allow meaningful subgroup analyses. No a priori sample size calculation was performed.

### 2.2. Variables and Definitions

The indications for 18F-FDG PET/CT were classified into four groups for all included patients: FUO, IUO, lymphadenopathy of unknown origin, and suspected solid mass [9,10]. In the field of infectious diseases, a significant proportion of radiological imaging studies are performed on patients with conditions such as sepsis and COVID-19. It is well established that the frequency of CT imaging increased during the pandemic [11]. A higher frequency of imaging is associated with a greater likelihood of detecting incidentalomas [12]. The suspected solid mass indication refers to incidentally detected solid lesions identified during the evaluation of patients for other clinical reasons, in whom 18F-FDG PET/CT was requested to clarify the etiology and guide further diagnostic workup. The interval between the initial clinical presentation and 18F-FDG PET/CT imaging was recorded in days.

Demographic and clinical variables included age, sex, body mass index (BMI), and time interval between the initial presentation and 18F-FDG PET/CT acquisition. Comorbid conditions that could affect the interpretation of the scans were documented. These conditions included diabetes mellitus, insulin use, chronic kidney disease, dialysis requirements, chronic liver disease, chronic pulmonary disease, chronic heart disease, HIV status, and immunosuppressive therapy [13].

The laboratory parameters recorded at presentation included white blood cell, neutrophil, lymphocyte, and platelet counts, ESR, and CRP levels. Microbiological findings included the presence of microorganisms in the blood cultures.

18F-FDG PET/CT-derived metabolic parameters included the maximum standardized uptake value (SUVmax), which was measured from the most hypermetabolic lesion concordant with the patient’s clinical symptoms and the presumed diagnostic focus.

Final diagnoses were recorded and categorized into five groups: infection, hematologic malignancy, solid organ malignancy, autoimmune/inflammatory disease, and benign masses. Final diagnoses were based on clinical follow-up, microbiological findings, histopathological evaluation, endoscopic assessment, molecular diagnostic methods, echocardiography, magnetic resonance imaging, and other advanced imaging modalities.

The diagnostic value of 18F-FDG PET/CT was defined as its ability to provide clinically meaningful results that support the final diagnosis. All 18F-FDG PET/CT examinations that met this definition were classified as diagnostic.

The primary outcomes of this study were the diagnostic yield of 18F-FDG PET/CT and its role in guiding histopathological biopsy decisions. The role of 18F-FDG PET/CT in guiding biopsy decisions and the clinical and metabolic factors associated with the need for biopsy were evaluated in this study.

### 2.3. Data Collection and Ethics

Patient data were obtained retrospectively from the hospital’s electronic medical record system and the nuclear medicine picture archiving and communication system (PACS), where all 18F-FDG PET/CT images were stored in the Digital Imaging and Communications in Medicine (DICOM) format. Owing to the retrospective observational design of the study and the use of anonymized data, informed consent was not required. This study was conducted in accordance with the principles of the 2013 Declaration of Helsinki, revised by the World Medical Association, and complied with the Non-Interventional Clinical Research Ethics Committee guidelines of the Kocaeli University Faculty of Medicine. The study was reviewed and approved under project number 2023/237, receiving ethical approval on 16 May 2025, with the approval code GOKAEK-2025/13/02.

### 2.4. 18F-FDG PET/CT Protocol

All patients fasted for a minimum of 4–6 h prior to undergoing imaging procedures. Before the radiotracer was injected, blood glucose levels were confirmed to be less than 200 mg/dL. The patients received an intravenous dose of 18F-FDG ranging from 4.5 to 14.5 mCi, which was adjusted based on body weight. After the injection, the patients rested in a quiet, dimly lit room for approximately 60 min before imaging.

18F-FDG PET/CT imaging was performed using a hybrid PET/CT scanner (Discovery 690; GE Healthcare, Chicago IL, USA). The patients were positioned supine and underwent whole-body imaging with a scanning field extending from the vertex to the feet. Low-dose CT scans were obtained for attenuation correction and anatomical localization (120 keV, 10–90 mAs, and a slice thickness of 2.5 mm). Subsequently, PET images were acquired in 3D mode with an acquisition time of approximately 2 min per bed position. The PET images were reconstructed using filtered back projection and repetitive reconstruction algorithms. The images were then reviewed in the axial, coronal, and sagittal planes.

### 2.5. Image Interpretation and Assessment

A nuclear medicine physician with extensive experience reviewed all the 18F-FDG PET/CT images. The physician was aware of the patients’ clinical presentations but was unaware of the final diagnosis. The primary basis for image interpretation was the visual assessment of abnormal focal or diffuse 18F-FDG uptake patterns that were inconsistent with physiological distribution.

The SUVmax was measured in the hypermetabolic lesion that was most clinically relevant, corresponding to the patient’s presenting symptoms and diagnostic focus. Areas of physiological uptake and degenerative changes were excluded. Then, 18F-FDG PET/CT findings were interpreted in conjunction with clinical, laboratory, microbiological, and other imaging data.

Scans were considered diagnostically contributory only when they suggested a specific etiologic diagnosis, such as spondylodiscitis, vascular infection, inflammatory disease, or malignancy [14]. This diagnosis was subsequently confirmed by microbiological, histopathological, radiological follow-up, serological, or clinical findings after a minimum follow-up period of six months.

According to the predefined study criteria, scans were classified as non-diagnostic when PET/CT demonstrated increased metabolic activity but could not reliably differentiate between infectious and malignant etiologies, and further diagnostic procedures such as biopsy were recommended in the official report. The recommendation for biopsy was documented only when it was clearly stated in the official PET/CT report. The non-diagnostic category included both indeterminate findings and cases in which PET/CT suggested a specific diagnosis that was not confirmed by the final outcome. Therefore, biopsy recommendation was inherently more common in the non-diagnostic group, as these cases required additional histopathological clarification to establish a definitive diagnosis.

### 2.6. Statistical Analysis

Statistical analyses were performed using IBM SPSS Statistics version 29.0 (IBM Corp., Armonk, NY, USA) and MedCalc version 14 (MedCalc Software, Ostend, Belgium). The normality of continuous variables was assessed using the Kolmogorov–Smirnov and Shapiro–Wilk tests. Continuous variables with a normal distribution were expressed as mean ± standard deviation (SD), whereas non-normally distributed variables were presented as median and interquartile ranges (IQR). Categorical variables were expressed as number and percentage. Comparisons between two independent groups were performed using the independent samples *t*-test or Mann–Whitney U test, as appropriate. The associations between categorical variables were analyzed using the chi-square test. Diagnostic performance indices (false positive, false negative, sensitivity, specificity, positive predictive value, negative predictive value, and accuracy) were calculated only for the binary outcome of infection versus no infection, as infectious diseases constituted the majority of the study cohort (58%). Agreement between 18F-FDG PET/CT-based infection classification and the final clinical diagnosis of infection was evaluated using Cohen’s kappa (κ) coefficient and interpreted according to the Landis and Koch criteria [15].

Factors associated with the need for histopathological biopsy were evaluated using multivariable logistic regression analysis. The variables were entered into the model in a stepwise manner based on the results of the univariable logistic regression analyses. Receiver operating characteristic (ROC) curve analysis was conducted to calculate the area under the curve (AUC) and to determine the optimal cut-off values, along with the corresponding sensitivity and specificity. A two-sided *p*-value < 0.05 was considered statistically significant for all analyses.

## 3. Results

From 2018 to 2023, 260 patients underwent 18F-FDG PET/CT scans as part of their evaluation by the Department of Infectious Diseases. After applying exclusion criteria (*n* = 74), 186 patients were included in the final study cohort of whom 87 were female and 99 were male (53%). The median age of the study population was 57 years old. The indications for 18F-FDG PET/CT and distribution of the final diagnoses are outlined in Table 1.

The demographic and clinical characteristics of the study population and the diagnostic contribution of 18F-FDG PET/CT are presented in Table 2. Patients with diagnostic PET/CT findings were older than those with non-diagnostic results (median 62 vs. 55 years, *p* = 0.007). PET/CT findings suggestive of infection were more frequent in the diagnostic group (64/109 [58.7%] vs. 1/77 [1.3%], *p* < 0.001). In contrast, biopsy was recommended more often in patients with non-diagnostic PET/CT results (43/77 [55.8%] vs. 43/109 [39.4%], *p* = 0.036). The isolated microorganisms in blood cultures included *Staphylococcus aureus* in four patients, *Streptococcus* species in four patients (*Streptococcus pneumoniae*, *Streptococcus mitis*, *Streptococcus sanguinis*, and *Streptococcus gordonii*), *Enterococcus faecium* (vancomycin-resistant) in one patient, and *Klebsiella pneumoniae* in one patient.

The benign mass group consisted of one sterile cardiac thrombus initially presenting as a cardiac mass, three pulmonary hamartomas, one focal nodular hyperplasia of the liver, and one dense-content hepatic cyst mimicking a mass lesion. None of these lesions demonstrated significant FDG uptake on PET/CT. Therefore, SUVmax values were not applicable for this subgroup.

Despite comprehensive clinical, microbiological, molecular, and imaging investigations during follow-up, no definitive diagnosis could be established in 10 patients (5%). These cases were retained in the analysis to reflect the complexity of real-world diagnoses.

The diagnostic yield of 18F-FDG PET/CT according to clinical indication is presented in Table 3, and no statistically significant difference was observed (*p* = 0.088). The diagnostic value of 18F-FDG PET/CT varied according to the final diagnosis category. A higher diagnostic yield was observed in malignant conditions, whereas a lower yield was noted in mycobacterial and autoimmune/inflammatory diseases (Table 4).

**Figure 1:**
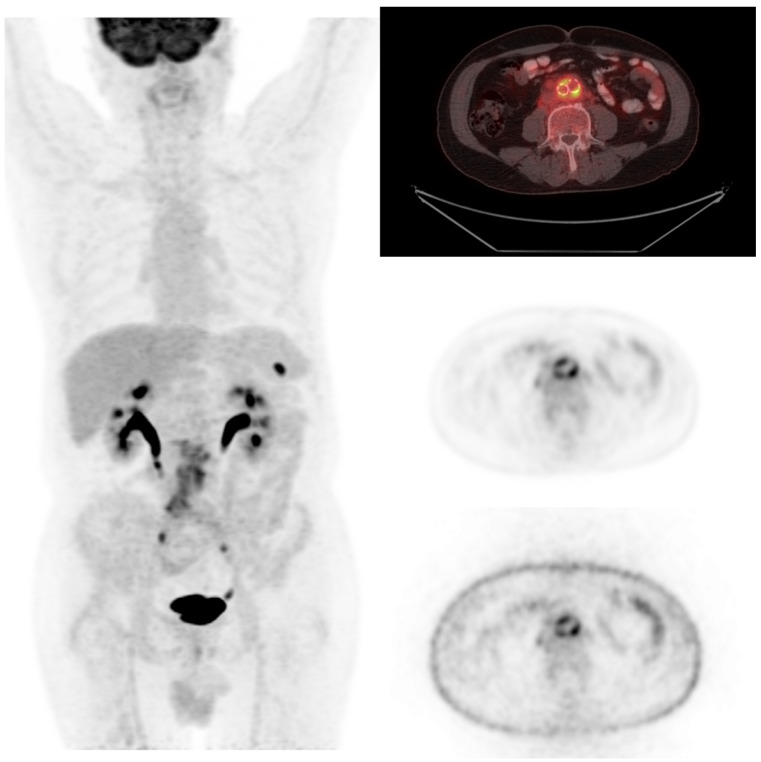
A 69-year-old male patient with a history of abdominal aortic graft placement underwent 18F-FDG PET/CT to evaluate fever of unknown origin. The scan revealed intense FDG uptake around the graft (SUVmax 9.1), indicating an aortic graft infection.

Among infectious diseases diagnosed using 18F-FDG PET/CT, bone and joint infections, including spondylodiscitis, and cardiovascular infections, such as infective endocarditis, had a particularly high diagnostic yield. Detailed numbers for all final diagnosis subgroups, including infectious, malignant, benign, autoimmune, and cases without definitive diagnosis, are presented in Table 5. As illustrated in Figure 1, a case of aortic graft infection was diagnosed using 18F-FDG PET/CT.

When patients with and without mycobacterial infections were compared, 18F-FDG PET/CT did not contribute to establishing a definitive diagnosis (*p* < 0.001). Furthermore, PET/CT more frequently recommended a histopathological biopsy in patients with a mycobacterial infection (15 out of 17 cases vs. 71 out of 169 cases, *p* < 0.001), and a biopsy was performed in all cases of mycobacterial infection (17 out of 17 cases vs. 85 out of 169 cases, *p* < 0.001).

SUVmax values were significantly higher in malignancies compared to infectious diseases (*p* < 0.001). However, there was no statistically significant difference in the SUVmax values between patients with malignancies and those with mycobacterial infections (*p* = 0.086). The mean SUVmax was 14.5 ± 7.2 in the malignancy group and 11.2 ± 4.6 in the mycobacterial infection group.

In contrast, patients with malignancy were significantly older, with a mean age of 64.2 ± 15.5 years, compared to 52.1 ± 17.1 years for patients with mycobacterial infection (*p* = 0.012). They also had a higher body mass index (28.3 ± 6.1 vs. 23.6 ± 4.5, *p* = 0.006). CRP levels also differed significantly between the groups (*p* = 0.020). The median CRP value was 111 mg/L (IQR: 35–209) in the malignancy group, compared with 37 mg/L (IQR: 11–91.5) in patients with mycobacterial infection.

When all patients were considered, 18F-FDG PET/CT findings suggestive of infection showed moderate diagnostic performance for identifying infectious etiologies. Among patients with a final diagnosis of infection (*n* = 107), PET/CT correctly identified infection in 64 cases (true positives) and failed to detect infection in 43 cases (false negatives), yielding a sensitivity of 59.8%. Among 79 patients without infection, PET/CT correctly excluded infection in 78 cases (true negatives) and incorrectly suggested infection in 1 case (false positive), corresponding to a specificity of 98.7%. The positive and negative predictive values were 98.5% and 64.5%, respectively, with an overall diagnostic accuracy of 76.3%. The degree of agreement between 18F-FDG PET/CT findings suggesting infection and the final diagnosis was moderate, with a Cohen’s kappa (κ) value of 0.547 (*p* < 0.001).

Given the limited diagnostic performance of 18F-FDG PET/CT in patients with mycobacterial infections, these cases were excluded from the secondary analysis. In the remaining cohort, the sensitivity of 18F-FDG PET/CT increased to 67.8%, whereas the specificity remained high at 98.7%. The positive and negative predictive values also improved, reaching 98.4% and 72.9%, respectively, with an overall diagnostic accuracy of 82.2%. In parallel, the agreement between the 18F-FDG PET/CT findings and the final clinical diagnosis strengthened to a substantial level (Cohen’s κ = 0.651, *p* < 0.001).

**Figure 2:**
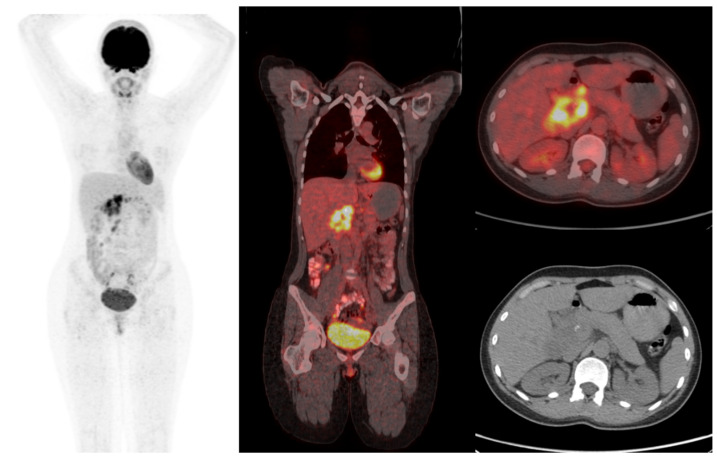
A 22-year-old previously healthy female evaluated for inflammation of unknown origin. Imaging by 18F-FDG PET/CT showed FDG uptake in the paraaortic, celiac, mesenteric, periportal, and perihepatic lymph nodes (SUVmax 15.4), raising suspicion for lymphoma. Subsequent biopsy confirmed *Mycobacterium tuberculosis* infection.

Figure 2 shows abdominal lymphadenopathy on 18F-FDG PET/CT in a patient who was initially evaluated for suspected lymphoma but was subsequently confirmed to have tuberculosis by PCR.

According to clinical indications, biopsies were most frequently performed in patients with lymphadenopathy. Among this group, the rates of biopsies and histopathological examinations were significantly higher than those in patients evaluated for FUO (*p* = 0.020) or IUO (*p* = 0.009).

Regarding the final diagnosis categories, biopsy was performed significantly more frequently in patients with hematologic and solid organ malignancies compared to those with infectious or autoimmune conditions (*p* < 0.01). Table 6 summarizes the clinical, laboratory, and PET/CT characteristics of patients who did and did not undergo biopsies.

**Figure 3:**
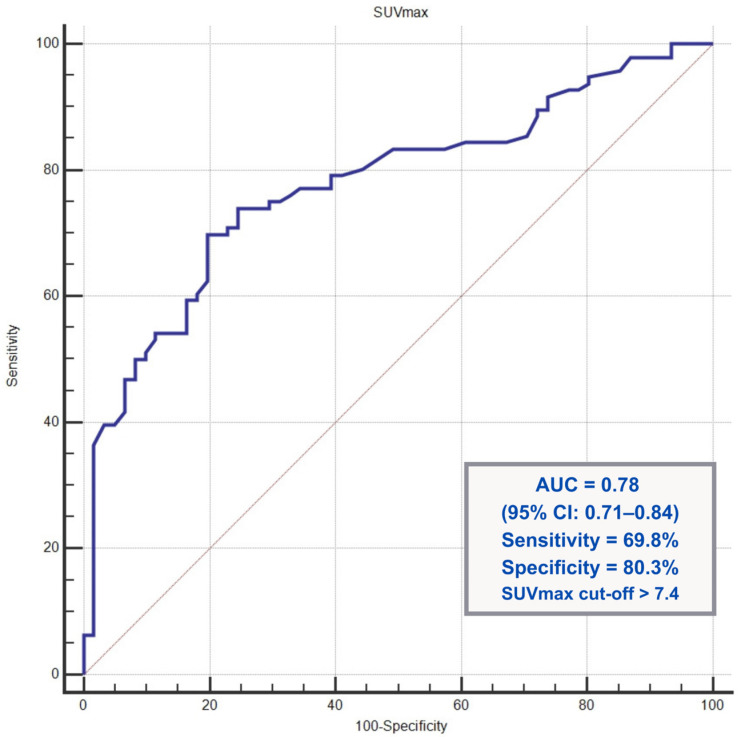
Receiver operating characteristic (ROC) curve of SUVmax for predicting biopsy performance following 18F-FDG PET/CT.

Because SUVmax values were significantly higher in patients who underwent biopsy, receiver operating characteristic (ROC) analysis was performed to evaluate the ability of SUVmax to discriminate between patients who did and did not undergo biopsy as part of real-world clinical decision-making. The analysis showed that SUVmax had a good ability to distinguish between patients who did and did not undergo biopsy, with an area under the curve (AUC) of 0.78 (95% confidence interval [CI]: 0.71–0.84; *p* < 0.001). An SUVmax threshold of >7.4 provided 69.8% sensitivity and 80.3% specificity for predicting the biopsy performance. The ROC curve is illustrated in Figure 3.

Multivariable logistic regression analysis revealed factors independently associated with biopsy and histopathological examination. These factors are listed in Table 7.

## 4. Discussion

In this retrospective single-center study, we evaluated 186 adult patients who underwent 18F-FDG PET/CT for diagnostic purposes in an infectious disease setting over a six-year period. Our findings demonstrate that 18F-FDG PET/CT provides clinically meaningful diagnostic value for differentiating between malignant and infectious conditions. The diagnostic yield of 18F-FDG PET/CT was particularly high for malignant conditions and non-mycobacterial infections, whereas its contribution was limited to mycobacterial and systemic autoimmune diseases. Biopsies were performed more frequently in patients with lymphadenopathy and higher SUVmax values when 18F-FDG-PET/CT findings did not suggest an infection. In light of these findings, this may be a valuable clinical tool for guiding histopathological biopsy decisions. We find that 18F-FDG PET/CT is a valuable diagnostic tool that aids in determining further management. This included indicating the need for a histopathological biopsy to differentiate suspected malignant lesions or support conservative management without biopsy when a benign or infectious process was suggested.

FUO and IUO remain diagnostically challenging conditions in clinical practice, despite significant advances in laboratory and imaging techniques. Their etiology encompasses a broad spectrum of conditions, including infections, malignancies, and non-infectious inflammatory diseases. This heterogeneity complicates the diagnostic processes. Consequently, 18F-FDG PET/CT has become a widely used imaging modality in the diagnostic evaluation of patients with FUO or IUO [16,17]. In our study, in line with the existing literature, FUO was the most common indication for 18F-FDG PET/CT, with 63 of 186 patients (34%) displaying it, followed by lymphadenopathy of unknown origin (27%) and inflammation of unknown origin (20%).

A recent review of the diagnostic performance of 18F-FDG PET/CT in patients with fever or inflammation of unknown origin found that PET/CT helped in the diagnosis in 59% of cases. Similarly, in our cohort, 18F-FDG PET/CT contributed to establishing the final diagnosis in 58.6% of the patients [17]. When the final diagnoses and diagnostic yield of PET/CT were evaluated, 18F-FDG PET/CT demonstrated the highest diagnostic yield in patients with malignancies. The diagnostic yield was 85.7% for hematologic malignancies and 88.9% for solid-organ malignancies. This is consistent with the existing literature [18]. Conversely, the lowest diagnostic yield (20.8%) was observed for autoimmune diseases. Within this group, 18F-FDG PET/CT was helpful in guiding the diagnosis of large vessel vasculitis, such as Takayasu arteritis, whereas it showed limited diagnostic value in systemic autoimmune diseases, such as systemic lupus erythematosus. Increased FDG uptake has been reported in treatment-naive large vessel vasculitis, which is consistent with our findings [19]. Similarly, FDG uptake patterns involving the bone marrow, spleen, and lymph nodes in systemic lupus erythematosus have been shown to be comparable to those observed in malignant conditions. This involvement may lead to false-positive interpretations of 18F-FDG PET/CT scans [20,21].

Overall, 18F-FDG PET/CT showed moderate diagnostic performance in identifying infectious etiologies, with a sensitivity of 59.8% and specificity of 98.7%. Although this modality showed a high ability to exclude infection, its sensitivity for detecting infectious causes was limited. The identified ratios were similar to those found in previous studies. Similarly, focal infections, including bone and joint infections and prosthetic endovascular infections, were also identified using 18F-FDG PET/CT in our study [22,23].

In our study, 18F-FDG PET/CT showed limited ability to differentiate mycobacterial infections from malignancies. Mycobacterial infections demonstrated FDG uptake patterns and SUVmax values similar to those observed in malignant diseases, which likely contributed to diagnostic uncertainty. Importantly, this limitation appears to be related not only to the infectious etiology itself but also to the clinical presentation, particularly in cases presenting with lymphadenopathy. It is known that 18F-FDG PET/CT is suboptimal in differentiating malignant from granulomatous lymphadenopathy, which may explain the observed diagnostic challenges [24]. Similar overlap in FDG uptake has also been reported in other inflammatory conditions such as sarcoidosis [25]. However, patients with mycobacterial infections were younger and had lower body mass indexes and CRP levels than those with malignancies. After excluding patients with mycobacterial infections, the sensitivity of 18F-FDG PET/CT for detecting infection increased to 67.8%, while the specificity remained high. The increased FDG uptake observed in active tuberculosis is believed to be indicative of the elevated metabolic activity exhibited by activated macrophages and lymphocytes. This heightened metabolic activity can result in SUVmax values that may resemble those seen in malignant lesions [26].

18F-FDG PET/CT plays an important role in guiding histopathological biopsy decisions, particularly when imaging findings raise suspicion of malignancy or remain inconclusive for infection. Biopsy procedures were performed more frequently in patients with lymphadenopathy and those with higher SUVmax values. In the regression analysis, PET/CT performed for lymphadenopathy was significantly and independently associated with biopsy (adjusted odds ratio [aOR] = 2.77, 95% confidence interval [CI] 1.09–7.07; *p* = 0.033). Similarly, PET/CT findings that were not suggestive of infection were strongly and independently associated with biopsy (aOR = 4.73, 95% CI, 2.07–10.82; *p* < 0.001).

Although there was a strong association between PET/CT-guided biopsy recommendations and actual biopsy performance, discrepancies were observed. These differences reflected real-world clinical complexity rather than intrinsic limitations of PET/CT. In certain infectious conditions, such as spondylodiscitis, a biopsy was performed to obtain microbiological cultures despite the absence of a PET recommendation. Conversely, in some cases where a biopsy was recommended by PET, invasive procedures could not be performed due to anatomical constraints (e.g., proximity to major vascular structures), technical limitations, or patient refusal. Additionally, in cases of lymphadenopathy with low SUVmax values, a biopsy was sometimes pursued due to hematological abnormalities or persistent clinical suspicion of malignancy. Similarly, in cases of inflammatory bowel disease, such as Crohn’s disease or CMV colitis, biopsy decisions were primarily driven by clinical and endoscopic findings rather than PET/CT results. These findings underscore the fact that, while PET/CT is an important adjunct in biopsy decision-making, it does not replace comprehensive clinical judgment.

High SUVmax values (>7.4) demonstrated the strongest association with biopsy and remained significant after multivariable adjustment (aOR = 4.98, 95% CI 2.11–11.76; *p* < 0.001). This analysis reflects clinical decision patterns rather than diagnostic accuracy. In contrast, lymphopenia was significant only in the univariable analysis (OR = 3.87, 95% CI 1.78–8.40; *p* < 0.001) and lost statistical significance in the multivariable model (aOR = 2.40, 95% CI 0.90–6.42; *p* = 0.080). This suggests that laboratory parameters had a more limited impact than PET/CT findings.

A review of lymphadenopathy in rheumatology practice emphasizes that in conditions such as rheumatoid arthritis, systemic lupus erythematosus, Sjögren’s syndrome, Castleman disease, and IgG4-related disease, prolonged lymphadenopathy requires careful exclusion of infection and malignancy. Nodal biopsy is recommended in cases of persistent diagnostic uncertainty [27]. According to the findings of the study by Kato et al., there was a comparison of reactive lymphadenitis and nodal lymphoma in patients with cervical lymphadenopathy. The mean SUV value was 12.8 for lymphoma nodes and 6.9 for reactive nodes. Higher SUV values and larger nodal sizes were associated with lymphoma [28]. Similarly, Broccoli et al. showed that a median SUVmax of 10.7 offered high diagnostic performance for lymphoma [29].

These findings align with those of the present study. In patients with prolonged lymphadenopathy, biopsy should be considered when SUVmax values are high and 18F-FDG PET/CT findings do not suggest an infectious etiology. Overall, these results suggest that 18F-FDG PET/CT can function as a decision-support tool. It facilitates the identification of patients requiring biopsy and aids in biopsy planning.

This study had several limitations. First, its retrospective and single-center design may limit the generalizability of our findings. The second, image interpretation was performed as part of routine clinical practice, and a formal assessment of inter-observer variability was not available. Finally, the number of patients in specific diagnostic subgroups, particularly those with mycobacterial and autoimmune diseases, was relatively limited.

Future studies should employ prospective, multicenter designs. Larger cohorts would enable more reliable subgroup analyses, particularly for autoimmune and heterogeneous conditions, such as mycobacterial infections. Additionally, structured reporting and artificial intelligence-assisted image analysis could reduce interobserver variability and improve reproducibility.

Despite these limitations, this study has several strengths. First, it reflects the real-world clinical practice in an infectious disease setting. Second, it included a relatively large and heterogeneous patient population with challenging diagnostic conditions. Additionally, this study evaluated PET/CT both as a diagnostic tool and as a decision support modality.

In conclusion, 18F-FDG PET/CT shows moderate overall diagnostic yield across infectious, inflammatory, and malignant conditions. Although its performance is limited in mycobacterial and systemic autoimmune diseases, it remains highly useful for guiding biopsy decisions, especially in patients with lymphadenopathy, elevated SUVmax values, and imaging findings that do not indicate infection. These findings suggest that 18F-FDG PET/CT is a useful tool for supporting clinical decision-making in challenging diagnostic scenarios.

## Figures and Tables

**Table 1:** Distribution of final diagnoses according to clinical indications for 18F-FDG PET/CT.

Final Diagnosis	FUO, *n*	IUO, *n*	LAP, *n*	Solid Mass, *n*	Total, *n* (%)	SUVmax, Median (IQR)
Infectious disease	33	23	34	17	107 (58)	5.9 (4.5–9.5)
Autoimmune disease	13	6	4	1	24 (13)	4.9 (3.3–8.2)
Hematologic malignancy	10	1	10	0	21 (11)	11.6 (8–19.9)
Solid organ malignancy	2	3	1	12	18 (10)	14 (11.3–19)
Benign masses	0	0	0	6	6 (3)	N/A
No definitive diagnosis	5	4	1	0	10 (5)	8.1 (4.4–13.7)
Total, *n* (%)	63 (34)	37 (20)	50 (27)	36 (19)	186 (100)	7.5 (4.6–11.5)

Abbreviations: 18F-FDG PET/CT: 18F-fluorodeoxyglucose positron emission tomography/computed tomography; FUO: Fever of unknown origin; IUO: Inflammation of unknown origin; LAP: Lymphadenopathy; SUVmax: The maximum standardized uptake value; N/A: Not applicable.

**Table 2:** Clinical and demographic characteristics of the study cohort according to 18F-FDG PET/CT diagnostic yield.

Variable	Total, *n* = 186	Diagnostic PET/CT, *n* = 109	Non-Diagnostic PET/CT, *n* = 77	*p* Value
Age (year), median (IQR)	57 (43- 71)	62 (50–73)	55 (38–69)	0.007 *
BMI (kg/m^2^), median (IQR)	26 (23–29)	26 (24–30)	25 (21–29)	0.077 ^†^
Sex				0.881 ^‡^
Female, *n* (%)	87 (47)	50 (46)	37 (48)	
Male, *n* (%)	99 (53)	59 (54)	40 (52)	
Presentation-to-PET/CT interval (days), median (IQR)	16 (8–27)	16 (9–28)	15 (7–27)	0.463 ^†^
Diabetes mellitus, *n* (%)	60 (32)	32 (29)	28 (36)	0.397 ^‡^
Insulin requirement, *n* (%)	22 (12)	10 (9)	12 (16)	0.270 ^‡^
Chronic kidney disease, *n* (%)	31 (17)	16 (15)	15 (20)	0.506 ^‡^
Dialysis requirement, *n* (%)	11 (6)	6 (6)	5 (7)	0.764 ^‡^
Chronic lung disease, *n* (%)	23 (12)	15 (14)	8 (10)	0.644 ^‡^
Chronic cardiac disease, *n* (%)	58 (31)	36 (33)	22 (29)	0.627 ^‡^
Chronic liver disease, *n* (%)	5 (3)	3 (3)	2 (3)	N/A
Immunosuppresive treatment, *n* (%)	10 (5)	6 (6)	4 (5)	1 ^‡^
Living with HIV	6 (3)	4 (4)	2 (3)	N/A
Blood culture positivity, *n* (%)	10 (5)	5 (5)	5 (7)	0.743 ^‡^
SUVmax, median (IQR)	7.5 (4.6–11.5)	6.4 (4.3–11.2)	8.6 (5.4–11.5)	0.063 ^†^
WBC (µL), median (IQR)	8715 (6392–11,963)	8570 (6590–11,900)	9300 (6305–12,900)	0.628 ^†^
Neutrophil (µL), median (IQR)	6080 (3985–9078)	5910 (3980–8600)	6884 (3980–9500)	0.454 ^†^
Lymphocyte (µL), median (IQR)	1500 (1000–2093)	1570 (900–2200)	1500 (1100–1980)	0.898 ^†^
Platelet (×10^3^/µL), median (IQR)	280 (205–367)	281 (195–339)	279 (210–378)	0.420 ^†^
CRP (mg/L), median (IQR)	75.5 (20–145)	80 (20–147)	71 (18–143)	0.545 ^†^
ESR (mm/h), median (IQR)	50.5 (25–70)	53 (22–72)	48 (26–69)	0.715 ^†^

Abbreviations: PET/CT: Positron emission tomography/computed tomography, IQR: Interquartile range, SUVmax: Maximum standardized uptake value, HIV: Human immunodeficiency virus, WBC: White blood cell count, CRP: C-Reactive protein, ESR: Erythrocyte sedimentation rate, N/A: Not applicable, * Independent *t*-test; ^†^ Mann–Whitney U Test, ^‡^ Chi-square test.

**Table 3:** Diagnostic yield of 18F-FDG PET/CT according to clinical indication.

Clinical Indication	Diagnostic PET/CT	Non-Diagnostic PET/CT
FUO, *n* (%)	31 (49.2)	32 (50.8)
IUO, *n* (%)	26 (70.3)	11 (29.7)
LAP, *n* (%)	27 (54.0)	23 (46.0)
Solid mass, *n* (%)	25 (69.4)	11 (30.6)
Total, *n* (%)	109 (58.6)	77 (41.4)

Abbreviations: 18F-FDG PET/CT: 18F-fluorodeoxyglucose positron emission tomography/computed tomography; FUO: Fever of unknown origin; IUO: Inflammation of unknown origin; LAP: Lymphadenopathy. Pearson chi-square test, *p* = 0.088.

**Table 4:** Final diagnosis categories and diagnostic contributions of 18F-FDG PET/CT.

Final Diagnosis Category	Diagnostic PET/CT, *n* (%)	Non-Diagnostic PET/CT, *n* (%)	Total	*p* Value
Infectious disease	64 (59.8)	43 (40.2)	107 (58)	0.764 *
Autoimmune disease	5 (20.8)	19 (79.2)	24 (13)	<0.001 *
Hematologic malignancy	18 (85.7)	3 (14.3)	21 (11)	0.015 *
Solid organ malignancy	16 (88.9)	2 (11.1)	18 (10)	0.013 *
Benign masses	6 (100)	0	6 (3)	0.043 *
No definitive diagnosis	0	10 (100)	10 (5)	<0.001 *
Total, *n* (%)	109 (58.6)	77 (41.4)	186 (100)	

Abbreviations: PET/CT: Positron emission tomography/computed tomography. * Chi-square test.

**Table 5:** Diagnostic Status and Biopsy Guidance of 18F-FDG PET/CT According to Final Diagnosis Subgroups.

Final Diagnosis	Total (*n*)	PET/CT Diagnostic (+), *n*	PET/CT Non Diagnostic (−), *n*	PET/CT Biopsy Advised (+), *n*	PET/CT Biopsy Advised (−), *n*	Biopsy Performed (+), *n*	Biopsy Performed (−), *n*
Infectious diseases							
Bacterial and viral infections	27	20	7	7	20	10	17
Bone and joint infections	17	13	4	7	10	10	7
Mycobacterial infections	17	3	14	15	2	17	0
Cardiovascular infections	6	6	0	0	6	0	6
Other infections (e.g., sepsis, abscesses, enteric fever, infectious colitis)	40	22	18	11	29	13	27
Autoimmune diseases							
Other autoimmune diseases	17	3	14	6	11	8	9
Large-vessel vasculitis	4	2	2	0	4	2	2
Sarcoidosis	3	0	3	3	0	3	0
Hematologic malignancies							
Lymphoma	14	13	1	13	1	14	0
Other hematologic malignancies	7	5	2	5	2	6	1
Solid organ malignancies	18	16	2	16	2	17	1
Benign masses	6	6	0	0	6	0	6
No definitive diagnosis	10	0	10	3	7	2	8

Abbreviations: PET/CT: positron emission tomography/computed tomography, LAP: Lymphadenopathy.

**Table 6:** Factors associated with performing biopsy and histopathological examination.

Variable	Biopsy (−), *n* = 84	Biopsy (+), *n* = 102	*p* Value
Age (year), mean ± SD	57.8 ± 16.6	56 ± 17.9	0.480 *
Sex			0.883 ^†^
Female, *n* (%)	40 (48)	47 (46)	
Male, *n* (%)	44 (52)	55 (54)	
Clinical indications for PET/CT, *n* (%)			0.002 ^†^
FUO	35 (42) ^a^	28 (28) ^a^	
IUO	23 (27) ^a^	14 (14) ^a^	
LAP	14 (17) ^b^	36 (35) ^b^	
Solid mass	12 (14)	24 (24)	
PET/CT recommended biopsy, *n* (%)			<0.001 ^†^
Yes, *n* = 86 (%)	11 (13.1)	75 (73.5)	
No, *n* = 100 (%)	73 (86.9)	27 (26.5)	
Final diagnosis category, *n* (%)			<0.001 ^†^
Infectious disease	57 (67.9) ^a^	50 (49.0)	
Autoimmune disease	11 (13.1)	13 (12.7)	
Hematologic malignancy	1 (1.2)	20 (19.6)	
Solid organ malignancy	1 (1.2)	17 (16.7)	
Benign masses	6 (7.1)	0	
No definitive diagnosis	8 (9.5)	2 (2)	
SUVmax, median (IQR)	5 (3.7–6.8)	9.8 (6.0–13.4)	<0.001
SUVmax > 7.4, *n* (%)	12 (19.7)	67 (69.8)	<0.001 ^†^
WBC (µL), median (IQR)	9700 (6832–12,225)	7805 (6168–11,663)	0.127 ^‡^
Neutrophil (µL), median (IQR)	6790 (4008–9159)	5600 (3928–8943)	0.400 ^‡^
Lymphocyte (µL), median (IQR)	1630 (1265–2228)	1300 (869–1862)	<0.001 ^‡^
Lymphopenia (<1000 µL), *n* (%)	10 (12)	35 (34)	<0.001 ^‡^
Platelet (×10^3^/µL), median (IQR)	306 (229–405)	263 (193–333)	0.011 ^‡^
CRP (mg/L), median (IQR)	85.5 (23–163)	68 (16–134)	0.338 ^‡^
ESR (mm/h), median (IQR)	57.5 (25–71)	49 (25–70)	0.748 ^‡^
PET suggested infection, *n* (%)	43 (51.2)	22 (21.6)	<0.001 ^†^

Abbreviations: PET/CT: Positron emission tomography/computed tomography, SD: Standard deviation, IQR: Interquartile range, * Independent *t*-test; ^†^ Chi-square test, ^‡^ Mann–Whitney U Test, FUO: Fever of unknown origin; IUO: Inflammation of unknown origin; LAP: Lymphadenopathy; SUVmax: The maximum standardized uptake. Different superscript letters indicate statistically significant differences between column proportions (Bonferroni-adjusted).

**Table 7:** Univariable and multivariable analysis of factors associated with biopsy and histopathological examination.

Variable	Univariable	*p*	Multivariable	*p*
	OR (95% CI)		aOR (95% CI)	
PET/CT performed due to LAP No (R)	2.73 (1.35–5.51)	0.005	2.77 (1.09–7.07)	0.033
PET not suggestive of infection Yes (R)	3.81 (2.02–7.21)	<0.001	4.73 (2.07–10.82)	<0.001
Lymphopenia (<1000/µL)	3.87 (1.78–8.40)	<0.001	2.40 (0.90–6.42)	0.080
High SUVmax > 7.4	9.43 (4.38–20.31)	<0.001	4.98 (2.11–11.76)	<0.001

Abbreviations: R: Reference OR: Odds ratio, aOR: Adjusted odds ratio, CI: Confidence interval, PET/CT: Positron emission tomography/computed tomography, LAP: Lymphadenopathy, SUVmax: The maximum standardized uptake.

## Data Availability

The authors will share the data and materials via e-mail upon request.

## Abbreviations

The following abbreviations are used in this manuscript:

FDG	Fluorodeoxyglucose
PET/CT	Positron emission tomography/computed tomography
STROBE	Strengthening the Reporting of Observational Studies in Epidemiology
FUO	Fever of unknown origin
IUO	Inflammation of unknown origin
BMI	Body mass index
SUVmax	Maximum standardized uptake value
PACS	Picture archiving and communication system
ESR	Erythrocyte sedimentation rate
CRP	C-reactive protein
DICOM	Digital Imaging and Communications in Medicine
IQR	Interquartile ranges
SD	Standard deviation
ROC	Receiver operating characteristic
AUC	Area under the curve
